# Old Age

**Published:** 1880-04

**Authors:** Thos. K. Beecher


### OLD AGE.

THOS. K. BEECHER.

If accurately estimated, no flash of lightning was ever as bright as ordinary sunshine. Yet when in the night the heavens are split from dome to base by one gash of blue-white dazzle, the eyes wince and the *seeming* is of a brightness far surpassing the noonday—"light as day," we say. If the sun at morning should flash up suddenly like midnight lightning, instead of coming after hours of broadening twilight, both man, bird and beast would shrink away and hide from the revealing outshine, the intolerable brightness. The brightness of the morning twilight!—did ever the watcher know precisely when the day began to break?

In like manner, at close of day, hath ever busy man or woman noted just when increase gives place to decrease of the light? Does the the traveler ever know just when the day is gone, and his journey begins to be a night-journey?

Each day is a life-time; and every life-time rightly viewed is but a day,—morning, noon and evening. Every night with its mystery of sleep, is to the thinker a death; and every morning a resurection to newness of life. The loss of consciousness during eight helpless hours of sleep, and the return to self and conscious identity, are as hopelessly beyond the power of physiology to explain satisfactorily, as is that "blessed hope" of resurrection—the comfort of every believer—yet, so often reckoned a mere phantasy by so called rationalists and men of science. Sleep and death have much in common. There's more than elegant poesy in the notion that a man may, and some men do so live, that when the evening comes they lie down content, as one who streaches himself to sleep in a familiar and delicious bed. Each day is a life-time, and the life of man is but his day.

The evening twilight of old age is a very delicious experience. It is so tender, so gradual, so harmonious and suggestive of perfect rest and refreshment. Of course the eye is to grow dim; for after the life-long hunger of the eye, and its greedy harvesting of countless sights, why should there not come to every man a time of review, adjustment, digestion and assimilation?—so that what has been seen with thoughtless eyes, may be re-visited by the eye of the mind and wrought into the texture of consciousness! He who has seen with childish eyes and cried, *oh! oh! just see that!* has not as yet *seen* at all. He has only looked at. That which abides with us is really seen.

It is well, therefore, that the eyes should grow dim, in order that the eye of the mind may see the better. No flower that grows is or can be so fair as the flower that grows, sheltered in the memory, never to fade. And if this closing in be a profit and a comfort, how delicious the slow imperceptible way of its coming. Who ever knew just when the perfect sight began to fail. It is a gathering twilight.

Again:—that which goes up must come down. Earth must back to earth, and the soul or life of a man must go upward. And when the evening draws near, the bones—our earthiest part, are the first to lose their toughness and elasticity of life; they can no longer endure the jumps and climbings, and fallings that come earlier in life. And yet they make no report of pain. They are not baulky; somehow the man no longer wants to jump or run as he once did. He who used to go at masts, steeples, towers and high chimneys, and climb them for fun of it, would now rather not go high, even if a baloon were at his service. The earth-love begins. And so, long before he knows it himself, he's walking and working and riding like an old man, contented, aye even glad to be old. Slow motions and quiet ways are so delicious. All this comes on so gradually that no man knows it or can know it, except by help.

Then too, as the juices that make young muscle and tissue plump and fresh, disappear, leaving many a line upon face and hands, it would seem as if these succulent refreshments of the first life had only retired a little within, to give freshness and sweetness to the thoughts of peace that accompany the age of him who has lived wisely. The appetites that have never been allowed to be masters, are now the gentle servants and obedient, of him whose inner man is a quickening toward eternal life to-morrow. The clamor and imperiousness of demand are silenced. A perfect choice and sovereignty, has come to the throne. The ripening saint is as one who has escaped out of a rushing panic of crazed crowds; he stands around the surging edges, hears the cries and curses, notes the blows, and blood and blasphemy, but somehow they no longer quicken, the fever throb of passion. He is sweetened; he cares not for argument, controversy, victory. What he knows or thinks he knows, is all his own. He recalls the costly years that have won it—and waits, for no man can hurry harvests.

This passing away of the combative temper, to be superseded by a gentle fatherly and motherly quality, is, like all the rest, as gradual as the coming of the bloom to the grape or the blush to the peach.

What a gentle sailing this voyage of life is—to be sure!

And then so gradually too, the social ties shift round, we never know just when. We called it bereavement at the time of it, and they kept a coming, until one day in that holy place where we meet in silent converse those whom we love, lo! it appears of a sudden, there are more of them ahead than there be around me. I am going to the general assembly of them all—I have treasure laid up.

A flower is not seen until you're done looking at it. A friend is not wholly and forever yours, until laid by in store.

Oh, but it is delicious to grow old, provided one have had wisdom to live thoughtfully, justly and reverently along the years of youth and manhood. The evening (of life) and the morning, (of resurrection) are man's first day. All before is at best chaos coming into order.

